# An Analysis of Patient Perceptions and Expectations to Dental Implants: Is There a Significant Effect on Long-Term Satisfaction Levels?

**DOI:** 10.1155/2017/8230618

**Published:** 2017-08-08

**Authors:** Shane J. J. McCrea

**Affiliations:** The Dental Implant and Gingival-Plastic Surgery Centre, Bournemouth, Dorset BH7 6AF, UK

## Abstract

Here we present an analysis of patient perceptions and expectations to dental implant placement and their prosthetic reconstruction, to then consider whether they have an effect on long-term satisfaction levels. A Post-Treatment Completion Questionnaire was designed to analyse whether patient satisfaction is influenced by age and/or gender; has an effect on patient-reported self-confidence levels; contributes to increased levels of oral hygiene; provides further insight into the average pain levels during and after the surgical intervention; or influences further acceptance of dental implant surgery. And then whether relationships exist between any of these factors. 182 consecutive patients completed the survey: 68 males and 114 females (age mean 64.68 years ± 11.23 SD); the average number of months since treatment completion was 37.4 (males) and 62.6 (females). There is a significant relationship between comfort rating and “how well informed” the patient was (*p* = 0.015). A significantly positive relationship exists between “considering dental implants in the future” and “overall experience” (*p* = 0.001). A significantly positive relationship exists between “overall satisfaction with appearance” and “satisfaction with comfort” (*p* = 0.011). A significant relationship exists between “overall satisfaction with appearance,” “satisfaction with comfort,” and “overall satisfaction with experience” (*p* = 0.001). The results amplify the need to transmit logical, truthful information to patients when dental implant treatment is being considered. The “fully informed” patient will have realistic expectations that lead to high degrees of satisfaction.

## 1. Introduction

Health care professionals accept that a direct relationship exists between a patients' anticipatory expectations of the end-goal of their treatment and the satisfaction level they experience when the treatment is achieved [1, 2]. Further, patient expectations towards treatment will predict their satisfaction levels as a result of treatment [3]. In a study evaluating patient expectations to the immediate-loading of two-implant-retained overdentures, 94.4% of the participants were found to be satisfied with their prosthesis in the very short-term, that is, 4 months, regardless of sociodemographic profiles and personality [4]. However, the authors felt that there is a need to understand patient expectations to help inform them as to treatment outcomes which would maximise their satisfaction. This view agreed with others who felt that patient misinterpretation of likely results leads to lower levels of satisfaction [5, 6].

Evaluation of dental implants encompasses aesthetics, functionality, longevity, and psychological parameters [7, 8]. Psychological aspects of success include aesthetics, comfort, function, hygiene, presentation, and satisfaction. Patient satisfaction includes self-esteem, self-concept, body-image, and Oral-Health-Related Quality of Life [9, 10]. Outcomes of single-tooth dental implants have been evaluated, but there is great variation in the collection and reporting methodology [11, 12]. Via telephone interviews, Moghadam et al. reported a range of 85–96% for patient satisfaction in aesthetics, comfort, and function for patients treated in a predoctoral implant program [13].

Today, “full informed consent” must precede interventional treatment, with patients being actively involved in the decision-making. Thus, in-depth questioning to evaluate and understand patient personal anticipated treatment goals is a prerequisite for end-point satisfaction. However, there is no accepted qualitative or quantitative method to accurately measure these expectations, since expectations encompass future concepts and the individual's psychological and physiological needs [9, 14]. Systematic reviews highlight the broadness present in the measures used to assess patient satisfaction levels. However, lack of consistency in the measurement methods will question their validity [1]. The date of review following treatment completion is at times minimal. It seems logical that to gain a thorough understanding of patient satisfaction, review dates should provide both short- and long-term assessments of patient satisfaction.

Other variables such as oral hygiene improvement, pain during and after treatment, or changes in self-confidence levels are not commonly reviewed. A more thorough analysis of these other variables should provide a more valid account of patient satisfaction following dental implant placement.

Today, with unprecedented advertising and the possibility of misleading information, patient awareness of dental implant therapy may increase but minimal experience of such a modality may result in unrealistic expectations [15, 16]. Therefore, use needs to be made of the known information providers to patients. An analysis of a random sample of 94 subjects found that a high percentage of respondents relied on the advice of friends, relatives, and neighbours when choosing a dentist [17], whilst it is also reported that the patients' dentist remains the principle source of dental implant information [18]. Additionally, other studies have demonstrated that quality of interpersonal communication between patient and healthcare provider significantly determines patient satisfaction levels [19, 20]. With such available information, it seems logical to nurture the development of a close patient/dentist professional relationship. This concept of enhancing such a relationship has been amplified in the study by Chaffin et al. They found that interpersonal experience had a strong association with a patient's assessment of their personal care and consequently their satisfaction levels [21]. In a similar study on a small cohort of 64 subjects, Cooper and Monson advocated 6- and 12-month follow-ups to confirm that satisfaction levels could be maintained [22].

Since patient satisfaction levels are directly associated with the levels of expectations predicted by the patient prior to a dental implant procedure, if expectations are not achieved, a negative influence on satisfaction levels ensues. It seems logical that such expectations are anticipated when considering the topic area of patient satisfaction since an intrinsic relationship exists between the psychological factors of self-reported confidence levels both prior to and subsequent to treatment and the psychological success of that treatment. The concept can be further enhanced by educating patients to fully understand the treatment modalities, the intricacies, and success rates together with a truthful discourse on visual, aesthetic, and physiological outcomes, so minimising overexpectation.

Difficulties can exist because Quality of Care criteria may be defined differently by the patient and implant surgeons [23]. In primary care, it is recognised that an association exists between patient perceptions, compliance with recommended treatment, satisfaction, and the resulting quality of healthcare and the eventual decision of the patient to return for further treatment [24].

The aim of this study is to contribute to existing knowledge and to review patient satisfaction following dental implant placement and prosthetic reconstruction. This study differs from others by focussing on the holistic perspective, incorporating both psychical and psychological outcomes. The patient cohort was large having varying time periods after surgical intervention and treatment completion. The analysis was to determine whether patient satisfaction would

- be influenced by age and/or gender;
- have an effect on patient-reported self-confidence levels;
- contribute to an increased level of oral hygiene;
- provide insight into average pain levels during the surgical intervention;
- influence further acceptance of dental implant surgery.

And then to analyse whether relationships exist between any of the above.

## 2. Materials and Methods

### 2.1. Study Design

Following a review of the literature, a trial Posttreatment Questionnaire was designed that incorporated the recorded commonly asked questions concerning patient satisfaction in conjunction with questions that specifically targeted responses to the dental implant surgery and the associative factors such as the impact on oral hygiene and self-confidence perception. This questionnaire was designed to comply with the statutory obligations required by the Care Quality Commission, UK, with regard to patient audit. A five-point Likert scale was utilised to measure the patient responses, providing patients with an effective response scale. Exclusion criteria were that the patients should not have had implant-retained dentures. The trial questionnaire was then presented to 20 consecutive patients. Following the trial, the reliability of the questionnaire was scrutinized using Cronbach's alpha coefficient; additionally, a text box was provided so that respondents had the opportunity to add further detail.

The definitive questionnaire was presented to all patients attending review appointments: no patient was in active treatment and they were in attendance for scheduled posttreatment reviews following completion of their restorative component. The survey spanned 9 consecutive calendar months during 2015-2016.

All patients had undergone dental implant replacement therapy. At consultation, if teeth had been deemed beyond further conservative therapy, the patients were provided with comprehensive treatment plans describing their individual presentation, treatment alternatives, proposed treatment, and the means by which treatment would be facilitated: being fully discussed in an ongoing manner offering patients reassurance and continual explanations as to the methodology and anticipated outcome in an attempt to avoid overexpectation and dissatisfaction. Effectively, completion of the post-active-treatment questionnaire was requested at varying postsurgery time-points. All patients were informed as to the use of the results and had given written informed consent.

The questionnaire was completed in privacy: no clinical staff were present, but nonclinical, reception staff were available for clarification of the questions. Where patients were uncertain of time periods, they were checked and recorded. Answers were recorded on computer and deidentified.

## 3. Results

One hundred and eighty-two consecutive patients attending for scheduled review appointments (all active treatment having been concluded) participated in this survey over a 9-month period: 68 males and 114 females (age mean 64.68 years ± 11.23 SD). The time from completion of treatment to the taking of the survey ranged from 16 months to 134 months. One patient declined to complete the questionnaire, producing a 99.45% completion rate. All patients had undergone dental implant replacement therapy that involved the placement of a single crown, multiple crowns, or fixed bridge-work; surgical areas included the anterior or posterior maxilla or mandible, or a combination of both. It is to be noted that the fixed prosthetic devices did not include dentures, whether full or partial.

### 3.1. Statistical Analysis

The satisfaction scores for each question in the questionnaire had been entered on a deidentification spread-sheet. Analysis of the results was carried out using Statistical Package for Social Science (IBM SPSS Statistics for Windows: Version 2015). Cronbach's alpha coefficient value was recorded as 0.87, inferring good internal consistency. Various statistical approaches were used. Statistical significance was assumed if the *p* value was <0.05.

The average number of months since the first implant was placed was 37.4 for males and 62.6 for females. Once the Levene's Test for Equality of Variances was assumed, no significant difference between genders was found: *t*(95) = −0.709, *p* = 0.480. The mean number of dental implants placed for both males and females was similar: male participants had a mean of 3.07 dental implants placed and females 3.05. Once Levene's Test for Equality of Variances had been assumed, a *t*-test showed no significant difference between the genders: *t*(177) = 0.052, *p* = 0.959. The majority of the patients (98.9%) felt that they were very well informed as to the procedures that would be carried out.

Using the five-point Likert scale, pain levels experienced by males and females was shown to be very similar: 4.64 and 4.54, respectively. The Levene's Test for Equality of Variances and an independent samples *t*-test were carried out, showing no significant difference for the satisfaction with the pain level responses between genders: *t*(177) = 0.793, *p* = 0.429.

The mean response for any oral health improvement as a result of the dental implant therapy was recorded as 4.81 (Likert scale), with no significant difference existing between genders: *t*(178) = 0.275, *p* = 0.783.

The relationship between self-confidence levels of patients and the position of the dental implants, that is, placed at the front of the mouth or to the side or both of these positions, was also assessed. For the front, the mean result was 4.32, for the side location, the result was 3.83, and for dental implants placed in both locations, the result was 4.22 on the Likert scale. An independent samples *t*-test investigated the significance between the differences of self-confidence levels dependant on the location of the dental implant. No significant difference existed when considering the location of the dental implant and self-confidence levels: *t*(176) = 1.539, *p* = 0.126. Further, it was found that there is no significant relationship between the overall satisfaction with appearance and the self-confidence levels of patients: *r* = 0.135, *n* = 177, and *p* = 0.074. Both genders indicated a mean response of “very satisfied” with the dental implant/crown appearance.

Table 1 shows a significant positive relationship between the crown satisfaction levels and the gum level satisfaction: *r* = 0.428, *n* = 162, and *p* = 0.001. However, no significant relationship was shown between the self-confidence levels and the gum level satisfaction and crown satisfaction: *r* = 0.148, *n* = 166, and *p* = 0.054.

Table 2 indicates a significantly positive relationship between the overall satisfaction with appearance and satisfaction with comfort: *r* = 0.464, *n* = 181, and *p* = 0.001. In addition, a significant relationship exists between comfort rating and the pain levels experienced: *r* = 0.190, *n* = 178, and *p* = 0.011.  Table 3 then goes on to show that a significant relationship exists between the comfort rating and how well informed the patient was: *r* = 0.181, *n* = 179, and *p* = 0.015. Additionally (Table 4), there is a significant relationship between the comfort rating and the overall experience of the patient: *r* = 0.301, *n* = 179, and *p* = 0.001.

When analysing a correlation between “considering dental implants in the future” and the participants “overall experience,” Table 5 shows that a significantly positive relationship exists between the two: *r* = 0.285, *n* = 173, and *p* = 0.001.

103 females (90.03%) and 63 males (92.65%) reported that they were very satisfied with the dental practitioner. One female patient (0.08%) reported herself as being very unsatisfied.  Table 6 indicates a significant relationship between recommending a friend for dental implants and the satisfaction with the dental practitioner: *r* = 0.387, *n* = 180, and *p* = 0.001. In addition, there is a significant relationship between the satisfaction with the dental practitioner and the self-confidence levels of the patient: *r* = 0.285, *n* = 176, and *p* = 0.001.

## 4. Discussion

The objective of this survey was to investigate whether a patient's level of satisfaction with implant-retained crown or bridge-work could be sustained over longer periods of time than that previously reported [4, 23]: males 37.4 months and females 62.6 months, with the recorded mean result for numbers of implants placed in males and females being very similar (3.07 and 3.05, resp.), showing no significant differences between the genders (*p* = 0.959). The questionnaire recorded and assessed the patient responses on a 5-point Likert scale ranging from 1 to 5, whereby the answers were determined by the type of question, ranging from

- very unsatisfied (1) to very satisfied (5);
- very insignificantly (1) to very significantly (5);
- very difficult (1) to very easy (5);
- extreme pain (1) to no pain at all (5);
- poorly informed (1) to very well informed (5).

The Likert scale had been chosen because of its ease of use. The Cronbach alpha coefficient was 0.87.

It could be postulated that, with time, a patient might become more critical concerning the “success” of their treatment, especially if there was a deterioration in crown-gingival harmony or where gingival recession had taken place and that recession is at a visible level; that is, perhaps a mandibular molar crown might not be considered as important, visually, as a maxillary incisor crown. If such deterioration was taking place, it could be speculated that there might be an adverse effect on a patient's perceived sense of self-confidence. In fact the results contradict this, recording no significant difference when considering the location of a dental implant and self-confidence levels (*p* = 0.126).

As stated, a patient's sense of satisfaction with the results of their implant surgery and prosthetic construction can be influenced by many factors: expectations as to the results, pain experienced during and after the surgery, the degree of preoperative information to allow “informed consent,” satisfaction with comfort, and overall satisfaction with appearance. The results showed that a very significant relationship exists between the overall satisfaction of appearance and satisfaction with comfort (*p* = 0.001): this implies that the positive response from patients experiencing low levels of pain and, therefore, higher levels of surgical comfort (intra- and postoperatively) will go on to produce a more positive attitude towards the end result of their treatment. The analysis also shows that gender influences the perceived comfort levels: there is a significant difference between male and females when considering levels of comfort with the dental implant procedure (*p* = 0.015), with males experiencing higher levels of comfort. These results amplify findings from other researchers. Epidemiological and clinical findings have clearly demonstrated that women are at increased risk for chronic pain and may well experience more severe clinical pain with studies [25] showing that women exhibit greater pain sensitivity, enhanced pain facilitation, and reduced pain inhabitation when compared to men [26].

Additionally, there is a significant relationship between the comfort rating and “how well informed” the patient was (*p* = 0.015). Full, informed consent should include the level of discomfort and pain a patient must expect to experience with dental implant treatment. If explained, their influence on comfort should be positive: the majority (98.9%) of the participants felt that they were very well informed. This was the partial goal of the treatment: it is being postulated that the fully informed patient would have preoperative information as to the anticipated levels of pain and discomfort.

This is further amplified, with the significant relationship found to exist between the overall satisfaction of appearance, comfort, and overall satisfaction with experience, *p* = 0.001. In addition, there is a very significant relationship between the comfort rating and the overall experience of the patient, *p* = 0.001. These very positive results would then explain the correlation between “considering dental implants in the future” and the participants' “overall experience”: a significantly positive relationship exists between them, *p* = 0.001.

Our results show that a significant relationship exists between the satisfaction with the dental practitioner and the self-confidence levels of the patient, *p* = 0.001 (see Table 6). This supports previous studies that demonstrated that the quality of interpersonal communication between patient and healthcare provider will determine the level of patient satisfaction with that care [19–21]. Our results go on to show that there is a very significant relationship between recommending a friend for dental implants and the satisfaction with the dental practitioner, *p* = 0.001.

The principle limitation of this study is that it was an internal audit in a single practice. Secondly, there was great variation in the time between the placement of the finished restorations on the implants and the completion of the questionnaire: 16 months–134 months.

## 5. Conclusion

Within the limitations of this study, it can be said that the analysed results indicate the need to transmit logical, truthful information to patients when dental implant treatment is being considered. The results indicate that the “fully informed” patient should have realistic expectations that lead to high degrees of satisfaction with the results that can be obtained with dental implant replacement therapy.

## Figures and Tables

**Table 1:** Correlations between crown satisfaction, gum level satisfaction, and self-confidence levels.

		Crown satisfaction	Gum level satisfaction	Self-confidence levels
Crown satisfaction	Pearson correlation	1	.428^*∗∗*^	.076
	Sig. (2-tailed)		.001	.329
	*N*	168	162	166

Gum level satisfaction	Pearson correlation	.428^*∗∗*^	1	.148
	Sig. (2-tailed)	.001		.054
	*N*	162	173	170

Self-confidence levels	Pearson correlation	.076	.148	1
	Sig. (2-tailed)	.329	.054	
	*N*	166	170	178

^*∗∗*^Correlation is significant at the 0.01 level (2-tailed). The table shows that there is a significant positive relationship between the crown satisfaction levels and the gum level satisfaction: *r* = 0.428, *n* = 162, and *p* = 0.001. There is no significant relationship between the self-confidence levels and the gum level satisfaction and crown satisfaction: *r* = 0.148, *n* = 166, and *p* = 0.054.

**Table 2:** Correlations between overall satisfaction of appearance, comfort, and pain levels.

		Overall satisfaction appearance	Overall comfort satisfaction	Pain levels
Overall satisfaction appearance	Pearson correlation	1	.464^*∗∗*^	.105
	Sig. (2-tailed)		.001	.165
	*N*	181	181	178

Overall comfort satisfaction	Pearson correlation	.464^*∗∗*^	1	.190^*∗*^
	Sig. (2-tailed)	.001		.011
	*N*	181	182	179

Pain levels	Pearson correlation	.105	.190^*∗*^	1
	Sig. (2-tailed)	.165	.011	
	*N*	178	179	179

^*∗∗*^Correlation is significant at the 0.01 level (2-tailed). ^*∗*^Correlation is significant at the 0.05 level (2-tailed). A significant relationship exists between the overall satisfaction of appearance and satisfaction with comfort: *r* = 0.464, *n* = 181, and *p* = 0.001. In addition, there is a significant relationship between the comfort rating and the pain levels: *r* = 0.190, *n* = 178, and *p* = 0.011.

**Table 3:** Correlations between overall satisfaction of appearance, comfort, and how well informed the patient felt.

		Overall satisfaction appearance	Overall comfort satisfaction	Well informed
Overall appearance satisfaction	Pearson correlation	1	.464^*∗∗*^	.066
	Sig. (2-tailed)		.001	.383
	*N*	181	181	179

Overall comfort satisfaction	Pearson correlation	.464^*∗∗*^	1	.181^*∗*^
	Sig. (2-tailed)	.001		.015
	*N*	181	182	180

Well informed	Pearson correlation	.066	.181^*∗*^	1
	Sig. (2-tailed)	.383	.015	
	*N*	179	180	180

^*∗∗*^Correlation is significant at the 0.01 level (2-tailed). ^*∗*^Correlation is significant at the 0.05 level (2-tailed). There is a significant relationship between the comfort rating and how well informed the patient was: *r* = 0.181, *n* = 179, and *p* = 0.015.

**Table 4:** Correlations between overall satisfaction of appearance, comfort, and overall satisfaction with experience.

		Overall satisfaction appearance	Overall comfort satisfaction	Overall experience
Overall satisfaction appearance	Pearson correlation	1	.464^*∗∗*^	.255^*∗∗*^
	Sig. (2-tailed)		.001	.001
	*N*	181	181	179

Overall comfort satisfaction	Pearson correlation	.464^*∗∗*^	1	.301^*∗∗*^
	Sig. (2-tailed)	.001		.001
	*N*	181	182	180

Overall experience	Pearson correlation	.255^*∗∗*^	.301^*∗∗*^	1
	Sig. (2-tailed)	.001	.001	
	*N*	179	180	180

^*∗∗*^Correlation is significant at the 0.01 level (2-tailed). A significant relationship exists between the comfort rating and the overall experience of the patient: *r* = 0.301, *n* = 179, and *p* = 0.001.

**Table 5:** Correlations between the consideration of dental implants in the future and the participants overall experience.

		Consider dental implants in the future	Overall experience
Consider implants in the future	Pearson correlation	1	.285^*∗∗*^
	Sig. (2-tailed)		.001
	*N*	173	173

Overall experience	Pearson correlation	.285^*∗∗*^	1
	Sig. (2-tailed)	.001	
	*N*	173	180

^*∗∗*^Correlation is significant at the 0.01 level (2-tailed). Here a significant positive relationship exists between the overall dental implant experience and the consideration of dental implants again in the future. *r* = 0.285, *n* = 173, and *p* = 0.001.

**Table 6:** Correlations between recommending a friend, satisfaction with dental practitioner and self-confidence levels.

		Recommend a friend	Satisfaction with dental practitioner	Self-confidence levels
Recommend a friend	Pearson correlation	1	.387^*∗∗*^	.258^*∗∗*^
	Sig. (2-tailed)		.001	.001
	*N*	180	177	176

Satisfaction with dental practitioner	Pearson correlation	.387^*∗∗*^	1	.064
	Sig. (2-tailed)	.001		.402
	*N*	177	177	174

Self-confidence levels	Pearson correlation	.258^*∗∗*^	.064	1
	Sig. (2-tailed)	.001	.402	
	*N*	176	174	178

^*∗∗*^Correlation is significant at the 0.01 level (2-tailed). The table indicates that there is a significant relationship between recommending a friend for dental implants and the satisfaction with the dental practitioner: *r* = 0.387, *n* = 180, and *p* = 0.001. In addition, there is a significant relationship between the satisfaction with the dental practitioner and the self-confidence levels of the patient: *r* = 0.285, *n* = 176, and *p* = 0.001.

## References

[1] Yao J., Tang H., Gao X.-L., McGrath C., Mattheos N. (2014). Patients' expectations from dental implants: A systematic review of the literature. *Health and Quality of Life Outcomes*.

[2] Crow R., Gage H., Hampson S. (2002). The measurement of satisfaction with healthcare: implications for practice from a systematic review of the literature. *Health Technology Assessment*.

[3] Jackson J. L., Kroenke K. (2001). The effect of unmet expectations among adults presenting with physical symptoms. *Annals of Internal Medicine*.

[4] Menassa M., de Grandmont P., Audy N., Durand R., Rompré P., Emami E. (2014). Patients’ expectations, satisfaction, and quality of life with immediate loading protocol. *Clinical Oral Implants Research*.

[5] Awad MA., Lund JP., Shapiro SH. (2003). Oral health status and treatment satisfaction with mandibular implant overdentures and conventional dentures. *Int J Prosthodont*.

[6] Heydecke G., Thomason J. M., Awad M. A., Lund J. P., Feine J. S. (2008). Do mandibular implant overdentures and conventional complete dentures meet the expectations of edentulous patients?. *Quintessence International*.

[7] Albrektsson T., Zarb G., Worthington P. (1986). The long-term efficiency of currently used dental implants: a review and proposed criteria of success. *The International Journal of Oral and Maxillofacial Implants*.

[8] Carr A., Wolfaardt J., Garrett N. (2011). Capturing patient benefits of treatment. *The International Journal of Oral & Maxillofacial Implants*.

[9] McGrath C., Bedi R. (2002). Population based norming of the UK oral health related quality of life measure (OHQoL-UK©). *British Dental Journal*.

[10] Ra'ed Omar A. H., Mahmoud Khalid A., Ahed Mahmoud A. (2006). Psychological impact on implant patients' oral health-related quality of life. *Clinical Oral Implants Research*.

[11] Belser U. C., Schmid B., Higginbottom F., Buser D. (2004). Outcome analysis of implant restorations located in the anterior maxilla: a review of the recent literature. *International Journal of Oral and Maxillofacial Implants*.

[12] Den Hartog L., Slater J. J. R. H., Vissink A., Meijer H. J. A., Raghoebar G. M. (2008). Treatment outcome of immediate, early and conventional single-tooth implants in the aesthetic zone: a systematic review to survival, bone level, soft-tissue, aesthetics and patient satisfaction. *Journal of Clinical Periodontology*.

[13] Moghadam M., Dias R., Kuyinu E., Ferguson M. B., Mucciolo T., Jahangiri L. (2012). Pre-doctoral fixed implant patient satisfaction and challenges of a clinical implant competency. *Journal of Dental Education*.

[14] Bowling A., Rowe G., Lambert N. (2012). The measurement of patients' expectations for health care: A review and psychometric testing of a measure of patients' expectations. *Health Technology Assessment*.

[15] Rustemeyer J., Bremerich A. (2007). Patients' knowledge and expectations regarding dental implants: assessment by questionnaire. *International Journal of Oral and Maxillofacial Surgery*.

[16] Pommer B., Zechner W., Watzak G., Ulm C., Watzek G., Tepper G. (2011). Progress and trends in patients' mindset on dental implants. I: Level of information, sources of information and need for patient information. *Clinical Oral Implants Research*.

[17] Alvesalo I., Uusi-Heikkila Y. (1984). Use of services, care-seeking behaviour, and satisfaction among university dental clinic patients in Finnland. *Community Dentistry and Oral Epidemiology*.

[18] Tepper G., Haas R., Mailath G. (2003). Representative marketing-oriented study on implants in the Austrian population. I. Level of information, sources of information and need for patient information. *Clinical Oral Implants Research*.

[19] Ben-Sira Z. (1980). Affective and instrumental components in the physician-patient relationship: an additional dimension of interaction theory.. *Journal of Health and Social Behavior*.

[20] Ross C. E., Duff R. S. (1982). Returning to the doctor: the effect of client characteristics, type of practice, and experiences with care.. *Journal of Health and Social Behavior*.

[21] Chaffin J. G., Chaffin S. D., Mangelsdorff A. D., Finstuen K. (2007). Patient satisfaction with dental hygiene providers in US military clinics. *Journal of dental hygiene*.

[22] Cooper B. R., Monson A. L. (2008). Patient satisfaction in a restorative functions dental hygiene clinic. *Journal of Dental Education*.

[23] Burke L., Croucher R. (1996). Criteria of good dental practice generated by general practitioners and patients. *International Dental Journal*.

[24] Gürdal P., Çankaya H., Önem E., Dinçer S., Yilmaz T. (2000). Factors of patient satisfaction/dissatisfaction in a dental faculty outpatient clinic in Turkey. *Community Dentistry and Oral Epidemiology*.

[25] Mogil J. S. (2012). Sex differences in pain and pain inhibition: multiple explanations of a controversial phenomenon. *Nature Reviews Neuroscience*.

[26] Bartley E. J., Fillingim R. B. (2013). Sex differences in pain: a brief review of clinical and experimental findings. *British Journal of Anaesthesia*.

